# Cannabidiol reduces oxycodone self-administration while preserving its analgesic efficacy in a rat model of neuropathic pain

**DOI:** 10.1038/s41598-025-31828-y

**Published:** 2026-01-11

**Authors:** Adriaan W. Bruijnzeel, Azin Behnood-Rod, Ranjithkumar Chellian, Wendi Malphurs, Robert M. Caudle, Marcelo Febo, Barry Setlow, Niall P. Murphy, John K. Neubert

**Affiliations:** 1 Department of Psychiatry, University of Florida, Gainesville, FL USA; 2 Center for Addiction Research and Education, University of Florida, Gainesville, FL USA; 3 Department of Orthodontics, University of Florida, Gainesville, FL USA; 4 Department of Oral and Maxillofacial Surgery, University of Florida, Gainesville, FL USA; 5 Department of Biomedical Sciences, Texas A&M University, Dallas, TX USA; 6 Department of Psychiatry, University of Florida, 1149 Newell Dr, Gainesville, FL 32611 USA

**Keywords:** Drug discovery, Health care, Medical research, Neuroscience

## Abstract

Prescription opioid misuse is a significant public health concern among individuals with chronic pain. Treating severe pain often requires high doses of opioids, increasing the risk of developing an opioid use disorder. Cannabidiol (CBD) is a non-intoxicating component of cannabis that has shown therapeutic potential without abuse liability. This study investigated the effects of CBD on oxycodone self-administration and hyperalgesia in an animal model of chronic neuropathic pain. Adult male rats were trained to self-administer intravenous oxycodone (0.06 mg/kg/infusion). Subsequently, they underwent chronic constriction injury (CCI) of the sciatic nerve or received sham surgery. Paw withdrawal latency was measured using the Hargreaves test as an indicator of thermal pain sensitivity. CBD (0, 1, 3, and 10 mg/kg, IP) was administered before the self-administration sessions, and pain testing was conducted afterward. The rats acquired oxycodone self-administration, as indicated by more active than inactive lever presses. CCI surgery decreased the paw withdrawal latency, confirming the induction of neuropathic pain. CCI alone did not affect oxycodone self-administration, suggesting that neuropathic pain does not substantially influence opioid intake at the dose tested. Treatment with CBD reduced oxycodone self-administration in both the sham and CCI rats. Oxycodone self-administration in the CCI rats reversed the CCI-induced decrease in paw withdrawal latency. However, CBD did not affect the antinociceptive effect of oxycodone in CCI rats. Taken together, these findings demonstrate that CBD reduces oxycodone self-administration without affecting the antinociceptive effects of oxycodone in neuropathic pain. This study supports the potential of CBD to reduce opioid use and misuse, regardless of pain status.

**Supplementary Information:**

The online version contains supplementary material available at 10.1038/s41598-025-31828-y.

## Introduction

The use of prescription opioids is widespread in the United States. It has been estimated that between 2019 and 2020, 12% of the general population used prescription opioids^1^. This prevalence increases among individuals with pain conditions. 30% of those with chronic pain and more than 40% of those with high-impact chronic pain report using prescription opioids^1^, highlighting that opioids continue to play a critical role in pain management. It has been estimated that 12% of the adults with an opioid prescription misuse opioids^2^. The motives for opioid misuse vary by age. Younger individuals often misuse opioids to experiment, relax, get high, or cope with negative emotions. In contrast, older adults typically misuse opioids for pain relief^3^. During the last two decades, there has been a strong increase in drug overdose deaths. The number of overdose deaths increased from fewer than 20,000 in 1999 to more than 100,000 in 2023^4,5^. The great majority of these deaths are caused by synthetic opioids^6,7^. Therefore, it is critical to develop treatment approaches with potential to reduce opioid use and misuse without compromising pain management.

Chronic use of opioids can lead to tolerance, physical dependence, and withdrawal symptoms upon discontinuation^8–10^. High-potency opioids like oxycodone are often used for their effective pain relief, but they also have a significant risk of abuse^11,12^. Oxycodone is a semi-synthetic opioid analgesic that is chemically derived from the naturally occurring opioid thebaine. Oxycodone activates mu, delta, and kappa 2b-opioid receptors and has a higher affinity for mu-opioid receptors (Ki value, 18 nM) than for delta (Ki value, 958 nM) and kappa-opioid receptors (Ki value, 677 nM)^13–15^. Oxycodone’s analgesic effects result from activating mu-opioid receptors in the brain, but stimulation of these receptors also causes euphoria, anxiolysis, and sedation^16–18^. Due to its potent efficacy for pain relief, oxycodone is widely used in clinical settings, but chronic use leads to tolerance and dependence. Given the high potential for misuse associated with oxycodone use for pain treatment, exploring adjunct therapies that could mitigate opioid misuse while preserving analgesia is of significant clinical interest.

Cannabis is widely used for the treatment of pain, but it also has psychoactive effects that produce abuse liability^19^. Cannabis contains a complex profile of cannabinoids, which have different pharmacological actions and therapeutic potential. Tetrahydrocannabinol (THC) is the main psychoactive constituent that produces the euphoric effects of cannabis and is responsible for its abuse liability^20^. Conversely, cannabidiol (CBD) exhibits a wide range of therapeutic properties, including analgesic, anti-inflammatory, anxiolytic, antidepressant, and anticonvulsant effects, but has no euphoric effects^21–27^. CBD exhibits complex pharmacology and acts through multiple mechanisms, interacting with cannabinoid receptors, transient receptor potential vanilloid 1 (TRPV1) channels, serotonin and GABA-A receptors, as well as opioid and dopaminergic receptor systems^28–30^. Notably, a purified form of CBD (Epidiolex^®^) has been approved for the treatment of seizures associated with Dravet syndrome, Lennox-Gastaut syndrome, and tuberous sclerosis complex^28,30^.

Both CBD and opioids such as morphine, fentanyl, and oxycodone have demonstrated analgesic effects in several preclinical models of chronic pain^27,31–39^. In addition, CBD has been shown to attenuate the reinforcing effects of opioids, including morphine, fentanyl, and oxycodone, in conditioned place preference and intravenous self-administration paradigms^40–42^. However, no studies have systematically examined the interaction between CBD and opioids on both reinforcement and analgesic properties under chronic pain conditions. Rivera-Garcia et al. reported that high-CBD cannabis vapor (64.2% CBD and 7.1% THC) attenuates fentanyl self-administration in rats with chronic pain^43^, but that study did not assess pain sensitivity in the CBD-exposed rats or test the effects of CBD alone. Similarly, Jesus et al. found that CBD enhances the analgesic effect of sub-effective morphine doses in rats with chronic pain but did not investigate opioid reinforcement under those conditions^32^. Collectively, these findings suggest that CBD may influence both the motivational and analgesic properties of opioids, raising the possibility that CBD could reduce opioid intake while maintaining or potentiating analgesic efficacy. Although recent preclinical work suggests that CBD vapor attenuates oxycodone reinforcement^41^, the effects of systemically administered CBD on both oxycodone reinforcement and analgesia under chronic pain conditions have not been examined. Building on these findings, the present study uniquely investigates the effects of systemically administered CBD on both oxycodone reinforcement and analgesia in the same animals, providing an integrated behavioral assessment under chronic neuropathic pain conditions. We hypothesized that CBD reduces oxycodone self-administration while maintaining or potentiating its analgesic efficacy in rats with chronic neuropathic pain. Our findings showed that CBD reduces opioid-reinforced responding while preserving the analgesic effects of oxycodone in animals with chronic neuropathic pain.

## Materials and methods

### Animals

Adult male Sprague Dawley rats (SD rats; 280–350 g; 8–9 weeks of age; *N* = 24) were purchased from Charles River (Raleigh, NC). The rats were kept in standard housing conditions (2 rats per cage) in a climate-controlled vivarium on a 12 h light-dark cycle (light off at 7 PM). The study was conducted during the light period of the light-dark cycle between 10 AM and 4 PM. Food and water were available ad libitum in the home cage throughout the study. The experimental protocols were approved by the University of Florida Institutional Animal Care and Use Committee (IACUC202107636). All experiments were performed in accordance with relevant guidelines and regulations of IACUC and in compliance with the ARRIVE guidelines 2.0 (Animal Research: Reporting of In Vivo Experiments).

### Drugs and treatment

For intravenous self-administration, oxycodone hydrochloride (NIDA Drug Supply Program) was dissolved in sterile saline (0.9% sodium chloride). The rats self-administered 0.06 mg/kg/infusion of oxycodone in a volume of 0.1 ml/infusion. The oxycodone dose is expressed as the weight of the salt. CBD (NIDA Drug Supply Program) was prepared in 5% ethanol and 5% Cremophor in PBS and administered intraperitoneally (IP) at a volume of 1 ml/kg body weight.

### Experimental design

**Fig. 1:**
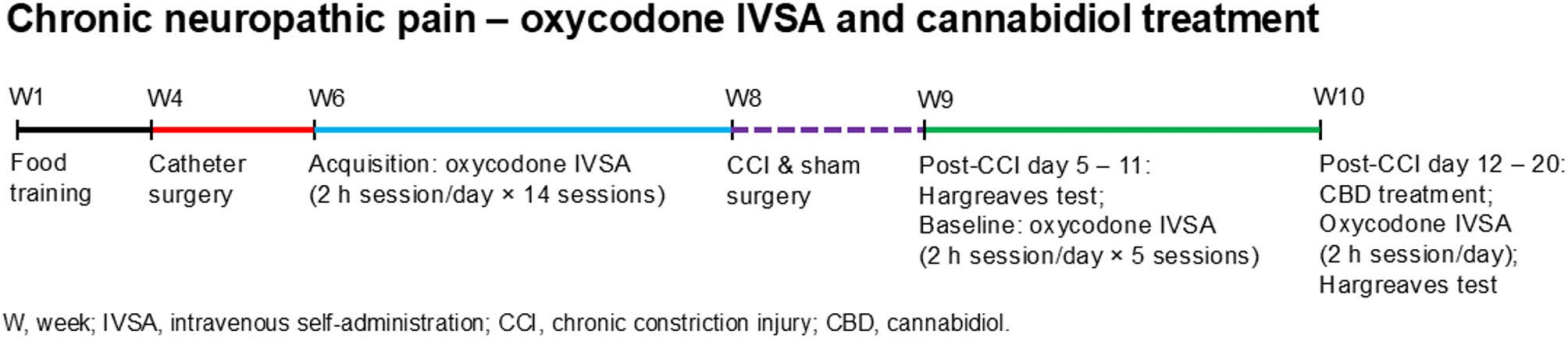
Schematic overview of the experiment. Adult male Sprague Dawley rats were trained to respond for food pellets. Following this, the rats were implanted with IV catheters and trained to self-administer oxycodone in 2-hour sessions per day for 14 sessions (*N* = 21). After the acquisition phase, chronic constriction injury (CCI) surgery was performed to induce neuropathic pain, while sham surgery was performed in control animals. Following recovery, the Hargreaves test was used to assess thermal pain sensitivity, and baseline oxycodone self-administration sessions were conducted in 2-hour sessions per day for 5 sessions. Subsequently, the effects of cannabidiol (CBD) treatment on oxycodone self-administration and thermal pain sensitivity were evaluated in both CCI and sham control rats. Sham, *N* = 11; CCI, *N* = 10. Abbreviation: IVSA, intravenous self-administration.

We investigated the effect of CBD on oxycodone self-administration in male rats under conditions of chronic pain induced by chronic constriction injury (CCI) and in non-pain (sham) states. A schematic overview of the experimental design is depicted in Fig. 1. Twenty-four rats were trained to press a lever for food pellets over a 10-day period. One week after the food training sessions, all rats underwent jugular vein catheterization surgery over 4 days to enable intravenous self-administration (IVSA) of oxycodone. Following surgery, the animals were given at least 7 days to recover. After recovery, a food reminder session was conducted using a fixed-ratio 1 (FR1) schedule with a 20-second time-out (TO20) during a 20-minute session to confirm that the rats retained lever-pressing behavior. The rats were then trained to acquire oxycodone self-administration (0.06 mg/kg/infusion) on an FR1-TO20 schedule during 120-minute sessions for 14 sessions. The unit dose of oxycodone (0.06 mg/kg/infusion) was selected based on previous work showing that this dose reliably maintains intravenous self-administration in rats and that CBD vapor exposure reduces oxycodone intake at the same dose under an FR1 schedule^41^. The left lever served as the active lever for 11 rats, while the right lever served as the active lever for 12 rats. The active lever assignment remained the same throughout the study. Responding on the active lever resulted in the delivery of an oxycodone infusion (0.1 ml infused over a 6.5-s period). The infusion was paired with a cue light, which remained illuminated throughout the time-out period. Responding on the inactive lever was recorded but did not have scheduled consequences. The active and inactive levers were retracted during the time-out period. Self-administration sessions were conducted five days per week. Rats reliably acquired oxycodone self-administration when they earned ≥ 15 infusions in two consecutive sessions. Following acquisition of oxycodone self-administration, rats were divided into two surgical groups: sham (*N* = 11) and CCI (*N* = 11). CCI surgery was performed to induce neuropathic pain, while sham surgery was performed to serve as a control. The CCI and sham groups were counterbalanced based on the left/right side of the active lever. In the CCI group, six rats self-administered oxycodone with the right lever as the active lever, while five rats used the left lever as the active lever. In the sham group, five rats self-administered oxycodone with right lever as the active lever, while six rats used the left lever as the active lever. Previous studies have shown that CCI of the sciatic nerve induces thermal hypersensitivity in adult male SD rats, as measured by the Hargreaves test, which remains relatively stable for at least one month post-surgery^44–47^. In this study, the Hargreaves test, oxycodone self-administration, and CBD treatment were conducted between post-CCI days 5 and 20. After the post-operative recovery period of four days, the Hargreaves test was conducted to assess thermal nociception as a measure of pain sensitivity in sham and CCI rats on post-CCI day 5. Baseline oxycodone self-administration sessions were then conducted for 5 days, with the Hargreaves test performed in the morning prior to the day 4 and 5 baseline self-administration sessions (i.e. on post-CCI days 10 and 11). Following the five-baseline oxycodone self-administration sessions, post-CCI day 12–20, CBD (0, 1, 3, and 10 mg/kg, IP) was administered according to a Latin square design, with 48 h separating successive CBD sessions. The doses of CBD were based on a previous study in which CBD pretreatment (0.3–30 mg/kg, IP) produced antinociceptive effects on mechanical sensitivity and thermal sensitivity to cold and heat in CCI rats^27^. CBD was administered 20 min before the 2 h oxycodone self-administration session to ensure systemic exposure during the session and to capture the ascending phase of absorption. In rats, CBD can be detected in plasma and the brain 30 min after intraperitoneal administration and reaches peak concentrations 60 to 120 min later^48–50^. In addition, behavioral studies have shown that CBD (3 mg/kg, IP) produces antinociceptive effects from 30 to 150 min post-injection in a rat model of postoperative incisional pain^51^. Therefore, immediately after the self-administration session (≈ 2 h 20 min post-injection), the Hargreaves test was performed to assess changes in pain sensitivity associated with CBD treatment and oxycodone intake, corresponding to the expected near-peak CBD exposure window. Although route-specific differences exist, the pharmacokinetic profile of intraperitoneal CBD in rats is broadly comparable to the delayed absorption phase of oral CBD in humans, which peaks at approximately 120 min following administration, supporting the translational relevance of the selected dosing interval^52^. Self-administration was also assessed in the intervening sessions (between successive CBD sessions). This experimental design allowed us to evaluate the effects of different doses of CBD on oxycodone intake in sham control rats and rats with chronic pain, as well as to assess any potential interaction effects of CBD and oxycodone on thermal hyperalgesia induced by CCI. One rat was excluded from the study after cathether implantation due to health issues. Additionally, one rat from the CCI group was excluded from the study due to excessively high responding on the inactive lever (> 200 inactive lever responses) across post-CCI self-administration sessions. During the self-administration period, catheter patency was tested by infusing 0.2 ml of the ultra-short-acting barbiturate Brevital (1% methohexital sodium). Rats with patent catheters displayed a sudden loss of muscle tone. If the rats did not respond to Brevital, their self-administration data were excluded from the analysis. On day 10 of oxycodone self-administration acquisition, one rat did not respond to Brevital, and its data were excluded from the study.

### Food training

Rats were trained to press a lever for food pellets in operant chambers placed in sound- and light-attenuated cubicles (Med Associates, St. Albans, VT) as in our previous work^53,54^. Food delivery was paired with a cue light, which remained illuminated throughout the time-out period. Twenty-four male rats were initially trained to respond for food pellets (F0299, 45 mg, chocolate-flavored pellets; Bio-Serv, Flemington, NJ) on a FR1 schedule using both levers over a period of 5 days. This was followed by an additional 5 days of training on an FR1 schedule with a 20-second time-out during 20-minute sessions using both levers. After completing the food training session on day 8, the rats were singly housed and remained singly housed for the remainder of the study. Three days before the start of the oxycodone self-administration sessions, the rats were allowed to respond for food pellets under the FR1-TO20 schedule during a single 20-minute session. Responding on both the right and left levers resulted in the delivery of a food pellet.

### Intravenous catheter implantation

The catheters were implanted as described before^55–57^. The rats were anesthetized with an isoflurane-oxygen vapor mixture (1–3%) and prepared with a catheter in the right jugular vein. The catheters consisted of polyurethane tubing (length 10 cm, inner diameter 0.64 mm, outer diameter 1.0 mm, model 3Fr, Instech Laboratories, Plymouth Meeting, PA). The right jugular vein was isolated, and the catheter was inserted 2.9 cm. The tubing was then tunneled subcutaneously (SC) and connected to a vascular access button (Instech Laboratories, Plymouth Meeting, PA). The button was exteriorized through a 1-cm incision between the scapulae. During the 7-day recovery period, the rats received daily infusions of the antibiotic gentamycin (4 mg/kg, IV, Sigma-Aldrich, St. Louis, MO). A sterile heparin solution (0.1 ml, 50 U/ml) was flushed through the catheter before and after administering the antibiotic and after oxycodone self-administration. After flushing the catheter, 0.05 ml of a sterile heparin/glycerol lock solution (500 U/ml, Instech Laboratories, Plymouth Meeting, PA) was infused into the catheter. The animals received carprofen (5 mg/kg, SC) daily for 72 h after the surgery.

### CCI surgery

Chromic gut suture (4.0, Ethicon) was cut into 2 cm pieces and immersed in sterile saline to prevent drying. Surgery was performed under aseptic conditions on a heating pad. Animals were administered buprenorphine (1.0 mg/kg, SC) before being maintained under general anesthesia (1–3% isoflurane in oxygen). The left hind leg was shaved and sterilized with chlorhexidine and 70% ethanol. A 5 to 7 mm incision was made in the skin below the femur and the skin separated from the muscle and connective tissue using blunt forceps. An incision was made through the femoris muscles, and the sciatic nerve freed using a glass pipette with a blunt curved tip. Three ligatures were made using a double knot 1 mm apart, tightened until the loop was just snug and the ligatures were unable to slide along the nerve. Silk sutures (5.0, Ethicon) were used to close the muscle layer and skin, and the wound was cleaned with chlorhexidine before application of triple antibiotic ointment. Rats in the sham control group underwent the same procedure (i.e., same paw, surgical intervention, treatment etc.) with the exception that no ligatures were placed.

### Hargreaves thermal paw withdrawal test

Animals were acclimated to a thermal hindpaw reflex testing apparatus (Ugo Basile model 7375a, Stoelting, Wood Dale, IL, USA) until they exhibited minimal spontaneous movement. A radiant heat lamp (intensity set at 45) was aimed at the left or right hind paw (random order) until the paw was withdrawn, with a cutoff time of 25 s to prevent tissue damage. The average of the first two latency measurements per paw, separated by approximately 2 min, was used for analysis.

### Statistics

Acquisition of oxycodone self-administration (14 sessions) data were analyzed using two-way ANOVAs with lever (active versus inactive) and session as within-subject factors. Oxycodone self-administration data (5 sessions) following sham and CCI surgery were analyzed using three-way ANOVAs with lever and session as within-subject factors, and surgery (sham versus CCI) as a between-subject factor. Hargreaves test data collected post-surgery, including assessments before the day 4 and 5 baseline oxycodone self-administration sessions, were analyzed using two-way ANOVAs with paw (right versus left) as a within-subject factor and surgery as a between-subject factor. Post-surgery, CBD treatment effects on oxycodone self-administration were analyzed using two-way ANOVAs with treatment as a within-subject factor and surgery as a between-subject factor. CBD treatment effects on Hargreaves test data (absolute paw withdrawal latency and percentage change from baseline) collected after oxycodone self-administration were analyzed using three-way ANOVAs with treatment and paw as within-subject factors, and surgery as a between-subject factor. The paw withdrawal latencies were expressed as a percentage of the drug-free baseline values obtained from the Hargreaves test conducted on post-CCI surgery days 10 and 11, prior to the initiation of CBD and oxycodone self-administration. For all statistical analyses, significant interaction effects identified in the ANOVAs were followed by Bonferroni’s post hoc tests to determine which groups differed. P-values less than or equal to 0.05 were considered significant. Data were analyzed using SPSS Statistics version 29 and GraphPad Prism version 10.1.2. Figures were generated using GraphPad Prism version 10.1.2.

## Results

### Acquisition of oxycodone self-administration

**Fig. 2:**
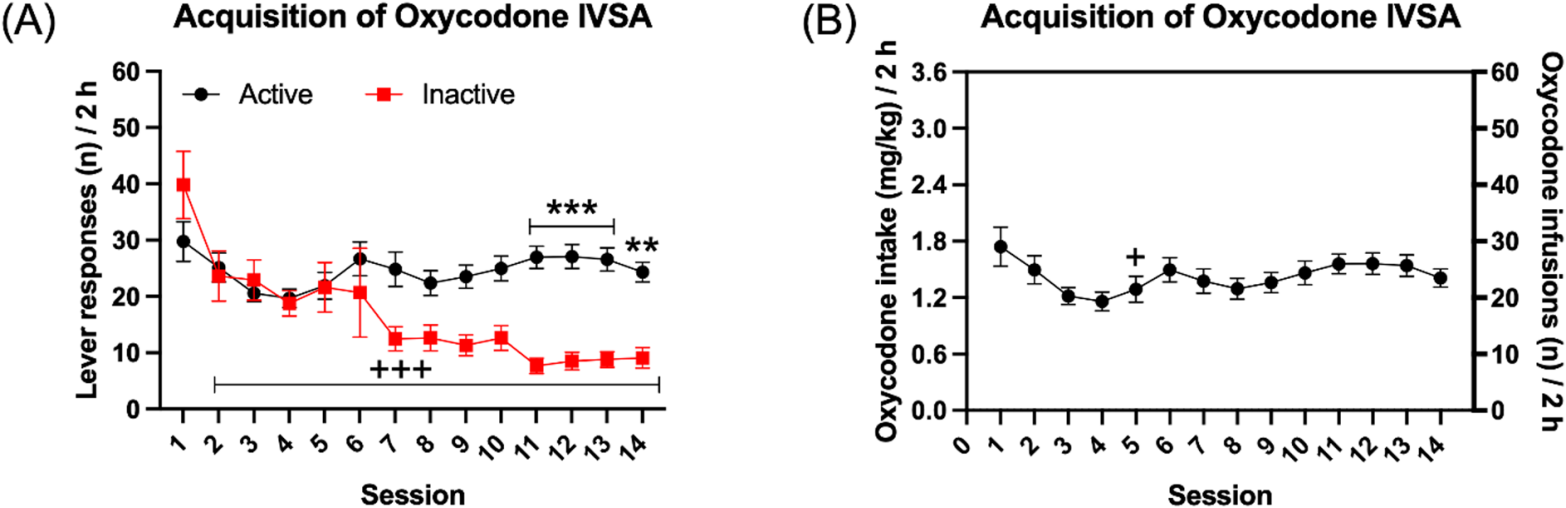
Acquisition of oxycodone self-administration in male rats. The rats were trained to respond for food pellets and then given access to oxycodone. The rats self-administered oxycodone for 14 sessions and active and inactive lever presses (**A**) and oxycodone intake (**B**) are shown. Asterisks indicate a significant difference in active lever responses and inactive lever responses during the same self-administration session. Plus signs indicate fewer inactive lever responses or lower oxycodone intake compared with the first self-administration session. +, *P* < 0.05; **, *P* < 0.01; +++, ***, *P* < 0.001. *N* = 21. Data are expressed as mean ± SEM.

The rats were initially allowed to self-administer oxycodone for 14 sessions. During this period, the rats responded more on the active lever than on the inactive lever and responding on the inactive lever decreased more over time than responding on the active lever (Fig. 2A, Lever F1,20 = 9.134, *P* < 0.01; Session F13,260 = 6.293, *P* < 0.001; Lever × Session F13,260 = 6.253, *P* < 0.001). The post hoc analysis revealed that the active lever responses were significantly higher than inactive lever responses from the eleventh session onward (Fig. 2A). In addition, inactive lever presses were significantly reduced from the second session onward compared to the first session (Fig. 2A). Furthermore, oxycodone intake initially decreased and then remained relatively stable over time (Fig. 2B, Session F13,260 = 4.072, *P* < 0.001). The post hoc analysis showed that oxycodone intake was significantly lower on the fifth session compared to the first session (Fig. 2B).

### CCI and hargreaves test

**Fig. 3:**
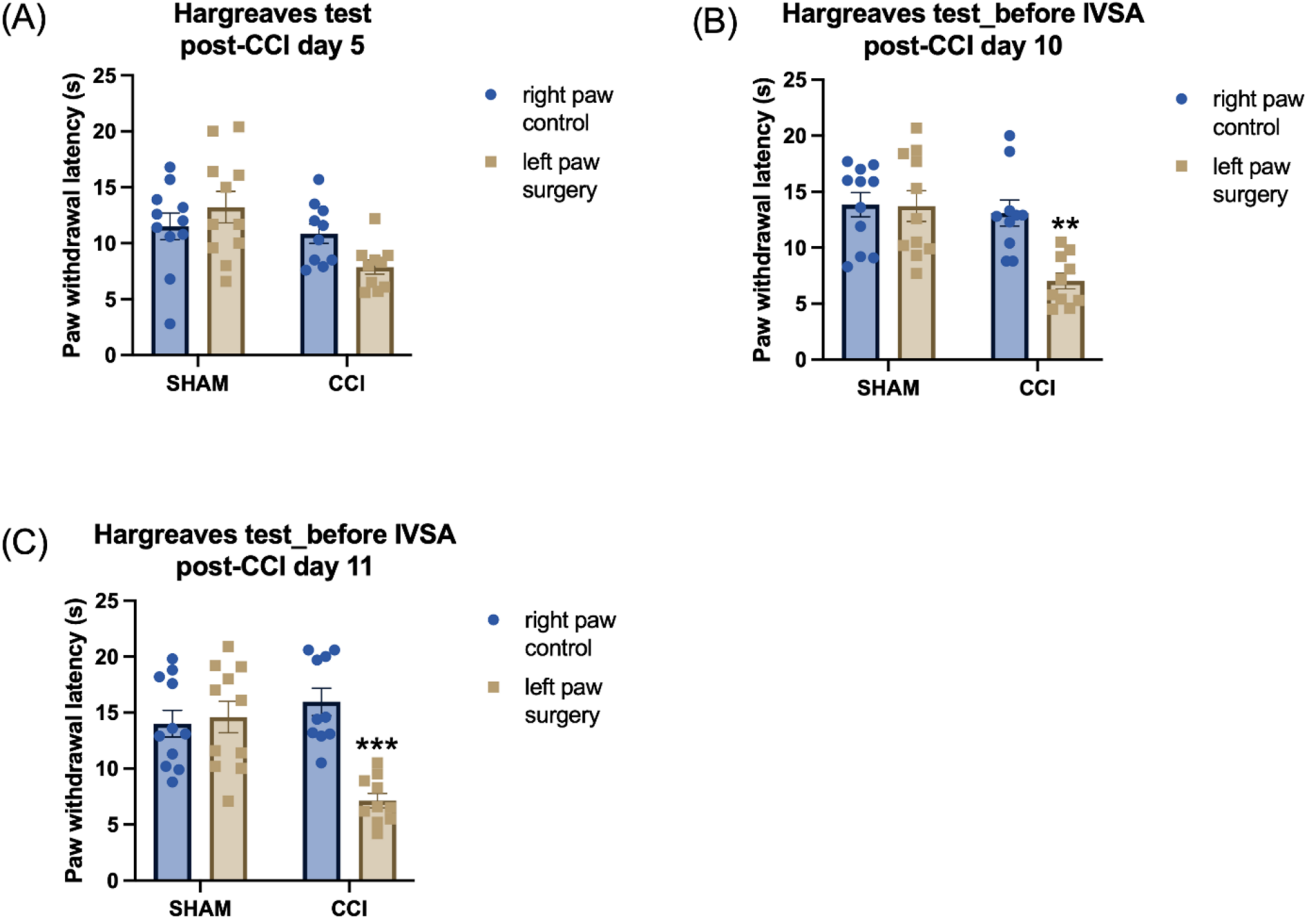
Paw withdrawal latencies are reduced in the Hargreaves test following CCI surgery. Thermal withdrawal latencies were measured in sham control and CCI rats on post-CCI day 5 (**A**), prior to the fourth (**B**, post-CCI day 10) and fifth (**C**, post-CCI day 11) baseline oxycodone self-administration sessions. Asterisks indicate lower paw withdrawal latencies in the ipsilateral (surgery) paw compared to the contralateral (control) paw in CCI rats, indicating increased thermal sensitivity in the Hargreaves test. **, *P* < 0.01; ***, *P* < 0.001. Sham, *N* = 11; CCI *N* = 10. Data are presented as mean ± SEM.

On post-CCI day 5, the CCI surgery decreased the paw withdrawal latency, and this effect was greater in the CCI paw than in the control paw (Fig. 3A, Surgery F1,19 = 5.583, *P* < 0.05; Paw F1,19 = 0.514, NS; Paw × Surgery F1,19 = 7.126, *P* < 0.05). However, the post hoc analysis did not reveal a significant difference in paw withdrawal latency between the control paw and surgery paw within either the sham or CCI groups (Fig. 3A). Furthermore, on post-CCI days 10 and 11 (before oxycodone self-administration sessions), withdrawal latency was shorter in the CCI paw compared to the paw that did not receive surgery and both paws of animals in the sham control group (Fig. 3B, post-CCI day 10: Surgery F1,19 = 7.872, *P* < 0.05; Paw F1,19 = 11.926, *P* < 0.01; Paw × Surgery F1,19 = 11.229, *P* < 0.01; Fig. 3C, post-CCI day 11: Surgery F1,19 = 4.475, *P* < 0.05; Paw F1,19 = 16.578, *P* < 0.001; Paw × Surgery F1,19 = 21.592, *P* < 0.001). On both these days, the post hoc analysis showed that only in CCI rats the paw withdrawal latency was significantly lower in surgery paw compared with control paw (Fig. 3B and C).

### CCI and oxycodone self-administration

**Fig. 4:**
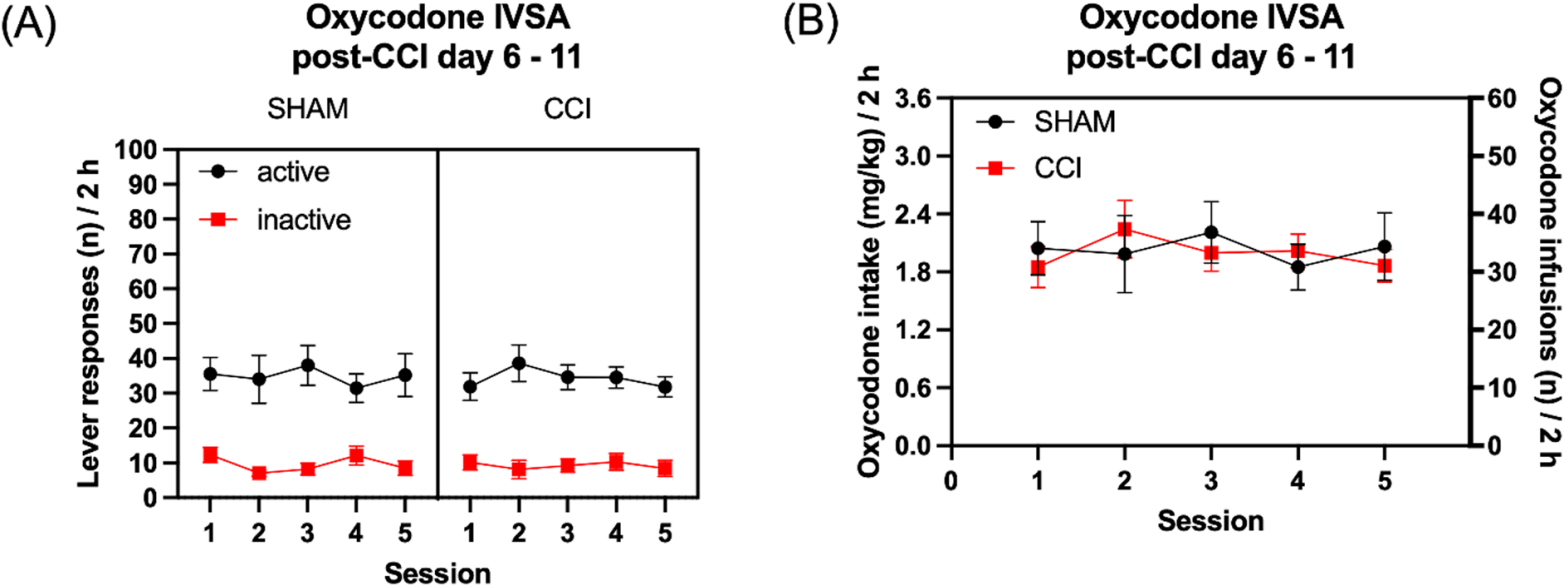
Neuropathic pain does not affect oxycodone self-administration in rats. The figures show oxycodone self-administration in sham control rats and CCI rats. The rats self-administered oxycodone for 5 days after the sham or CCI surgery and active and inactive lever presses (**A**) and oxycodone intake (**B**) are shown. Sham, *N* = 11; CCI *N* = 10. Data are expressed as mean ± SEM.

The CCI surgery did not affect responding on the active or the inactive lever, and the rats continued to respond more on the active than the inactive lever (Fig. 4A, Surgery F1,19 = 0.026, NS; Lever F1,19 = 43.123, *P* < 0.001; Lever × Surgery F1,19 = 0.001, NS; Session F4,76 = 0.466, NS; Session × Surgery F4,76 = 1.524, NS; Lever × Session F4,76 = 2.677, *P* < 0.05; Lever × Session × Surgery F4,76 = 1.228, NS). The CCI surgery also did not affect oxycodone intake (Fig. 4B, Surgery F1,19 = 0.010, NS; Session F4,76 = 1.077, NS; Session × Surgery F4,76 = 1.778, NS).

### Effects of CBD pretreatment on oxycodone reinforcement and analgesia

#### Oxycodone self-administration (2 h) conducted 20 min after CBD pretreatment

**Fig. 5:**
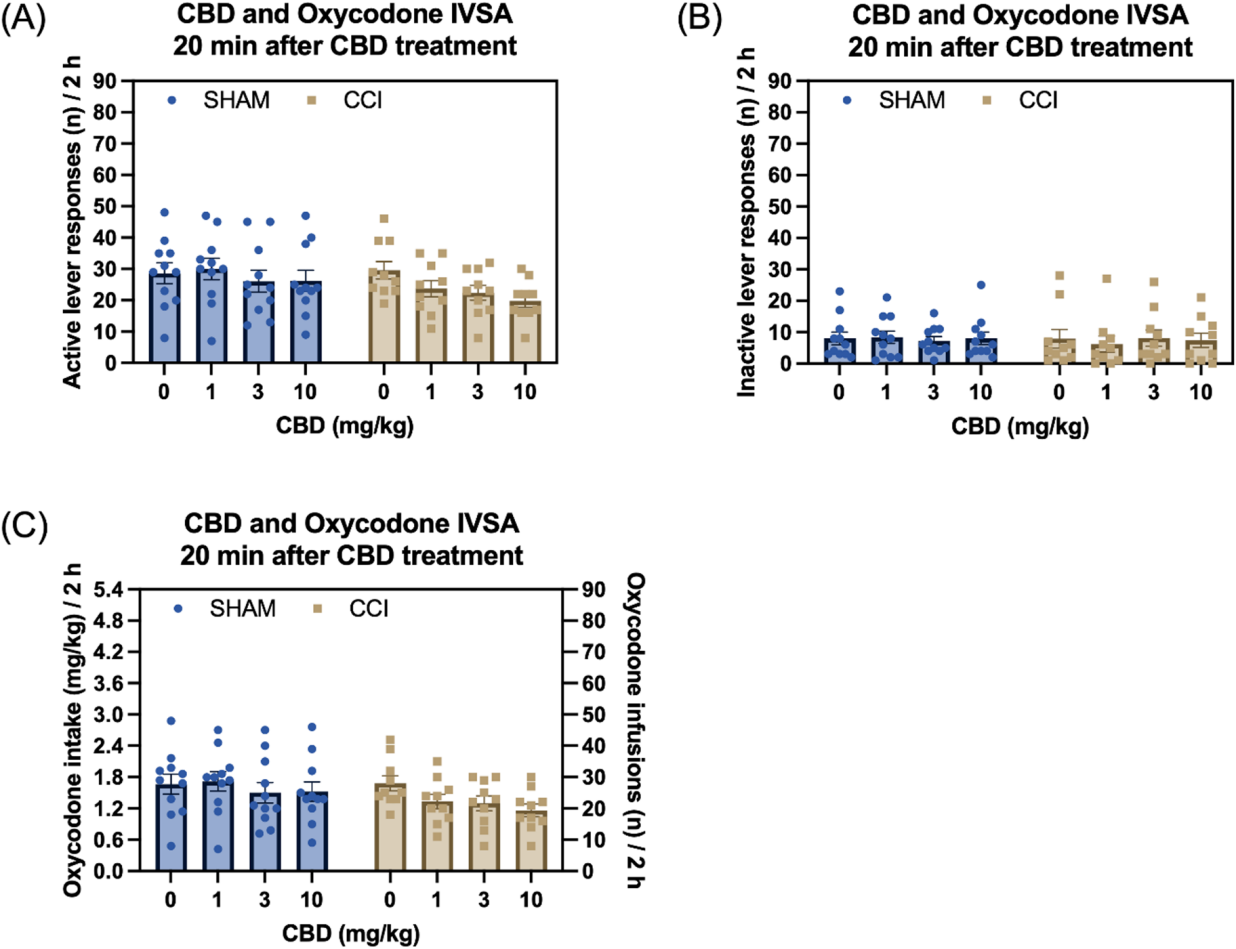
CBD treatment reduces oxycodone intake in both sham control and CCI rats. Treatment with CBD reduced active lever responses in both the CCI and sham control groups, with no effect of CCI surgery on active lever responses or the response to CBD (**A**). Neither CBD treatment nor CCI surgery affected inactive lever responses (**B**). CBD treatment decreased oxycodone intake in both the CCI and sham control groups, while CCI surgery had no effect on oxycodone intake (**C**). Sham, *N* = 11; CCI *N* = 10. Data are presented as mean ± SEM.

Treatment with CBD decreased responding on the active lever in both the sham control and CCI group and the magnitude of this reduction did not differ between the two groups (Fig. 5A, Surgery F1,19 = 1.072, NS; Treatment F3,57 = 5.151, *P* < 0.01; Treatment × Surgery F3,57 = 2.045, NS). Neither CBD nor surgery condition affected inactive lever responses (Fig. 5B, Surgery F1,19 = 0.033, NS; Treatment F3,57 = 0.109, NS; Treatment × Surgery F3,57 = 0.556, NS). CBD also decreased oxycodone intake to an equivalent extent in both surgical conditions (Fig. 5C, Surgery F1,19 = 1.266, NS; Treatment F3,57 = 4.614, *P* < 0.01; Treatment × Surgery F3,57 = 1.832, NS).

#### CBD and oxycodone-induced antinociception (Hargreaves test)

##### Absolute paw withdrawal latency

**Fig. 6:**
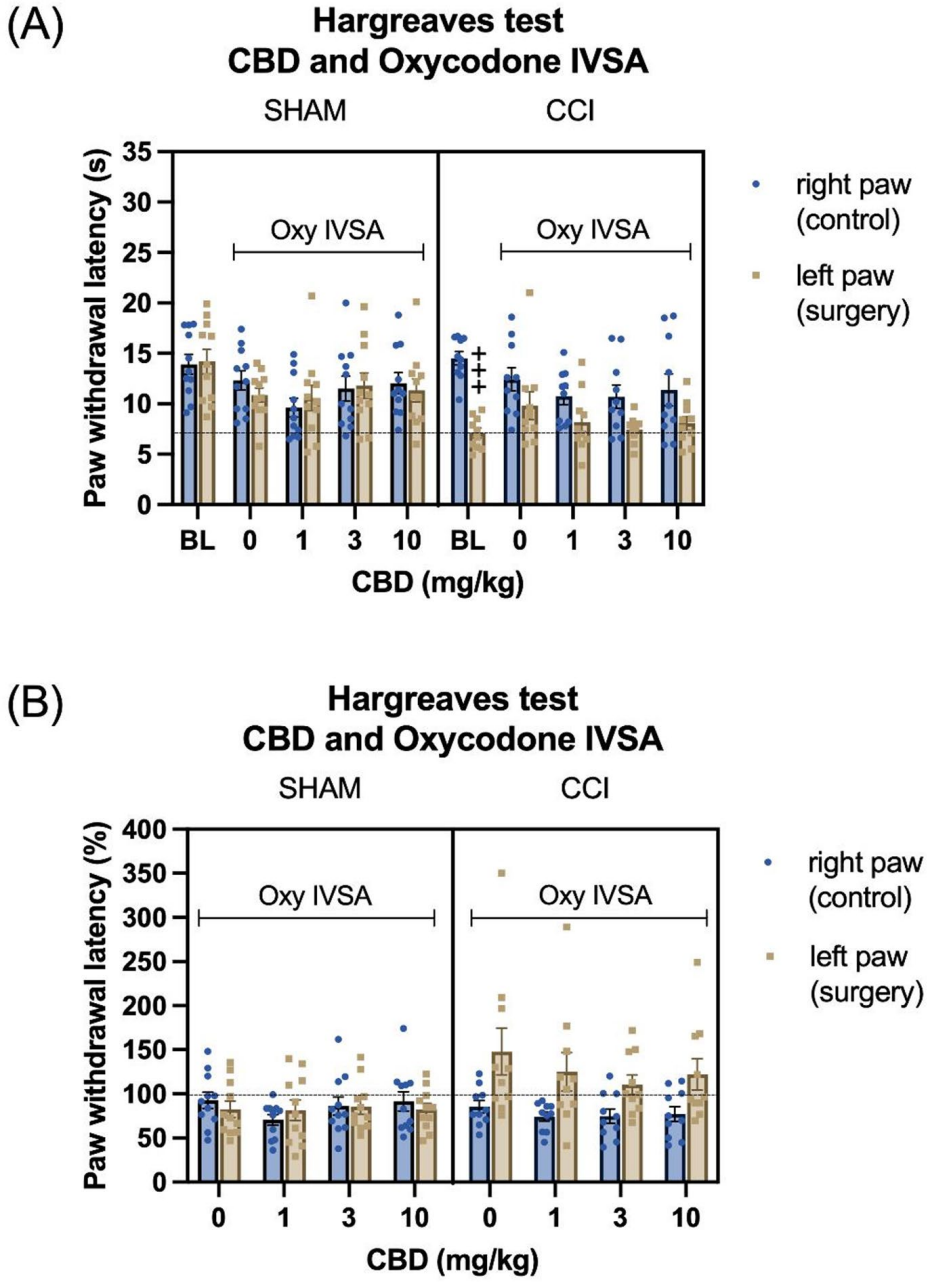
CBD treatment did not alter the antinociceptive effect of oxycodone self-administration in CCI rats. The Hargreaves data are shown as absolute paw withdrawal latency (A) and as percentage change from baseline (B). Baseline (BL) values represent drug-free conditions (i.e., in the absence of CBD or oxycodone) and were obtained as the average of post-CCI days 10 and 11. At baseline, CCI surgery reduced paw withdrawal latency in the ipsilateral (surgery) paw compared to the contralateral (control) paw, indicating increased thermal sensitivity in the Hargreaves test (A). In vehicle-treated CCI rats (i.e., in the absence of CBD), oxycodone self-administration increased paw withdrawal latency in the CCI paw relative to the contralateral paw, as reflected in the percentage change from baseline, indicating an antinociceptive effect (B). However, treatment with CBD in combination with oxycodone self-administration did not alter the paw withdrawal latency in the CCI paw compared to the contralateral paw (A and B). Plus signs indicate significantly lower absolute paw withdrawal latencies in the ipsilateral paw compared with the contralateral paw in CCI rats at baseline. BL, baseline; Oxy, oxycodone. +++ *P* < 0.001. Sham, *N* = 11; CCI *N* = 10. Data are presented as mean ± SEM.

In drug-free condition (baseline - average of post-CCI day 10 and 11 Hargreaves testing conducted without CBD or oxycodone on board), CCI surgery decreased paw withdrawal latency in the surgery paw (Surgery F1,19 = 5.541, *P* < 0.05, Paw F1,19 = 17.54, *P* < 0.001; Paw × Surgery F1,19 = 15.388, *P* < 0.001). However, immediately following oxycodone self-administration, this reduction in latency was reversed in both vehicle and CBD treated CCI rats (Fig. 6A; Treatment F4,76 = 3.475, *P* < 0.05; Treatment × Surgery F4,76 = 1.219, NS; Treatment × Paw F4,76 = 1.84, NS; Treatment × Paw × Surgery F4,76 = 2.597, *P* < 0.05). The post hoc analysis revealed that the significant reduction in withdrawal latency in the surgery paw compared with the control paw was only observed during the baseline drug-free condition in CCI rats. In addition, post hoc analysis showed that oxycodone self-administration had no effect on paw withdrawal latency in either vehicle or CBD treated sham rats.

##### Percentage change in paw withdrawal latency from baseline

To assess the relative changes from baseline data (average of post-CCI day 10 and 11 Hargreaves testing conducted without CBD or oxycodone on board), paw withdrawal latencies were expressed as percentage change (Fig. 6B). In CCI rats, oxycodone self-administration increased paw withdrawal latency in surgery paw (Fig. 6B, Surgery F1,19 = 3.113, NS; Paw F1,19 = 7.8, *P* < 0.05; Paw × Surgery F1,19 = 9.484, *P* < 0.01). CBD treatment did not alter the effect of oxycodone self-administration on paw withdrawal latency in the CCI surgery paw (Fig. 6B, Treatment F3,57 = 1.62, NS; Treatment × Surgery F3,57 = 1.007, NS; Treatment × Paw F3,57 = 0.601, NS; Treatment × Paw × Surgery F3,57 = 0.962, NS).

### Oxycodone self-administration conducted 24 h after CBD treatment

During the self-administration sessions interspersed between CBD test sessions, neither treatment with CBD nor CCI condition affected responding on the active lever (Fig. S1A, Surgery F1,19 = 0.003, NS; Treatment F3,57 = 1.44, NS; Treatment × Surgery F3,57 = 0.408, NS) or the inactive lever (Fig. S1B, Surgery F1,19 = 0.517, NS; Treatment F3,57 = 0.422, NS; Treatment × Surgery F3,57 = 0.511, NS). There was also no effect of CBD treatment or CCI surgery on oxycodone intake (Fig. S1C, Surgery F1,19 = 0.117, NS; Treatment F3,57 = 1.469, NS; Treatment × Surgery F3,57 = 0.103, NS).

## Discussion

This study tested the hypothesis that CBD would reduce oxycodone self-administration while maintaining or potentiating its analgesic efficacy in a model of chronic neuropathic pain. Consistent with this hypothesis, CBD pretreatment reduced oxycodone intake without diminishing oxycodone’s antinociceptive effects in CCI rats. The rats acquired oxycodone self-administration as indicated by more lever presses on the active than the inactive lever. The CCI procedure reduced the paw withdrawal latency in the Hargreaves test, confirming the development of thermal hyperalgesia, a hallmark of neuropathic pain. However, CCI alone did not alter oxycodone self-administration. Treatment with CBD significantly reduced oxycodone intake in both sham and CCI rats. Notably, oxycodone self-administration produced antinociceptive effects in CCI but not sham rats. Together, these findings suggest that CBD reduces oxycodone self-administration regardless of pain status while preserving its antinociceptive effects in an animal model of chronic neuropathic pain.

CCI of the sciatic nerve is a well-established model of chronic neuropathic pain in rodents^44–47,58,59^. In this study, CCI decreased the withdrawal latency in the Hargreaves test, indicating that CCI induces thermal pain hypersensitivity, a feature of chronic neuropathic pain. Interestingly, we observed no effect of CCI surgery on oxycodone self-administration, which contrasts with some previous reports. For instance, it has been reported that spinal nerve ligation of the L5 and L6 dorsal nerve roots reduces opioid (heroin, morphine, fentanyl, hydromorphone, and methadone) self-administration in rats^60^. Similarly, rats with spared nerve injury, which involves ligation of the left tibial and common peroneal nerves while sparing the sural nerve, show reduced fentanyl self-administration compared to sham-operated controls^43^. Furthermore, a study using a chronic inflammatory pain model (intraplanar injection of Complete Freund’s Adjuvant, CFA) found that pain modulated how heroin affected dopamine release in the nucleus accumbens and heroin self-administration, with these effects being dose-dependent^61^. A low dose (0.075 mg/kg, IV) of heroin reduced dopamine release, whereas a high dose (0.15 mg/kg, IV) enhanced dopamine release. Inflammatory pain did not affect the self-administration of 0.05 and 0.1 mg/kg/infusion of heroin but increased the intake of the higher dose (0.2 mg/kg/infusion)^61^. Taken together, these studies suggest that different types of opioids and the doses of opioids used in chronic pain conditions may differently affect the self-administration of opioids. In the current study, we examined the effects of neuropathic pain on oxycodone self-administration using a single dose (0.06 mg/kg/infusion) in CCI rats. However, previous studies have shown that rats readily self-administer higher doses of oxycodone^62,63^. Therefore, further investigation is warranted to determine whether higher doses of oxycodone may reveal different effects of neuropathic pain on oxycodone self-administration in rats.

In this study, CBD treatment reduced oxycodone intake in both sham and CCI groups. This finding that CBD reduces oxycodone intake aligns with previous studies exploring the effects of CBD on opioid intake. For example, Nguyen et al. demonstrated that acute CBD vapor inhalation decreases oxycodone self-administration, suggesting that CBD may reduce the rewarding properties of oxycodone^41^. Our results also align closely with those of Rivera-Garcia et al., who demonstrated that high-CBD cannabis vapor (64.2% CBD and 7.1% THC) reduces fentanyl self-administration in both spared nerve injury and sham-operated female rats^43^. Furthermore, both intraperitoneally administered CBD and inhalation of high-CBD whole-plant cannabis extract prevents the development of morphine-induced conditioned place preference in mice and rats^40,43^. In addition, CBD blocked the reward-enhancing effect of morphine in the intracranial self-stimulation procedures in rats^64^. Inhaled CBD also prevented fentanyl-induced conditioned place preference in mice^42^. Importantly, preliminary clinical findings indicate that CBD (Epidiolex^®^) reduces cue-induced craving in individuals with opioid use disorder who are treated with buprenorphine^65^. Taken together, these preclinical and clinical observations suggest that CBD decreases the rewarding properties of opioids and may help mitigate opioid misuse irrespective of pain status.

In this study, oxycodone self-administration in vehicle-treated rats increased the paw withdrawal latency in the CCI paw but not in sham rats, suggesting that oxycodone mediated antinociceptive effects only in neuropathic pain conditions. Notably, both sham and CCI rats self-administered similar amounts of oxycodone per session (~ 1.6 mg/kg total intake/session), raising the possibility that the lack of effect in the sham rats may be related to insufficient total drug exposure. A previous study reported that oxycodone self-administration at a comparable unit dose (0.06 mg/kg/infusion) produced antinociceptive effects in SD rats in the tail-flick assay^66^. However, the total oxycodone intake in that study was higher (~ 2.7 mg/kg over a 3-hour session) compared to the intake in our sham group (~ 1.6 mg/kg over a 2-hour session)^66^. This difference in cumulative intake may be critical, as the median effective dose (ED₅₀) for antinociception following subcutaneous administration of oxycodone in male rats is approximately 1.7 mg/kg, with full antinociception observed at 5.6 mg/kg^67^. Similarly, the ED₅₀ following intraperitoneal administration is about 1.46 mg/kg, with full antinociception at 4 mg/kg^68^. Unlike a single bolus injections, intravenous self-administration results in cumulative drug exposure that depends on session duration and intake behavior. Therefore, it is possible that the total oxycodone intake in sham rats was below the threshold required for measurable antinociceptive effects. In contrast, the same dose was sufficient to alleviate thermal hypersensitivity in CCI rats, suggesting that pain state modulates the efficacy of oxycodone self-administration.

We did not examine the effects of CBD alone on thermal pain sensitivity in drug-naïve (e.g., saline self-administration) sham or CCI rats. However, a previous study reported that sub-chronic intraperitoneal administration of CBD (0.3–30 mg/kg) on post-CCI days 22–24 produced antinociceptive effects in the hot plate assay in male CCI rats^27^. In the present study, CBD treatment (1–10 mg/kg) did not affect the antinociceptive effects of oxycodone self-administration in CCI rats. An important alternative interpretation is that, because CBD reduced oxycodone intake in CCI rats, the preserved antinociceptive effect may reflect a pharmacodynamic interaction between CBD and oxycodone, whereby CBD’s intrinsic antinociceptive properties could partially offset the reduction in oxycodone exposure. This possibility highlights a key limitation of the current study and underscores the need to evaluate the effects of CBD alone on nociception in CCI rats. In contrast, in an acute pain model, previous research has shown that CBD blocked the antinociceptive effect of oxycodone in the hot plate test in mice^69^. Furthermore, CBD in combination with morphine produced subadditive effects on morphine-induced antinociception in the hot plate assay^70^. Notably, in CCI rats, intraperitoneal CBD produced anti-allodynic effects in the Von Frey mechanical sensitivity test only at the highest dose tested (30 mg/kg), but not at lower doses (3 and 10 mg/kg)^32^. Moreover, combining the effective (30 mg/kg) and subeffective (10 mg/kg) doses of CBD with subeffective doses of morphine resulted in anti-allodynic effects only when 30 mg/kg CBD was included^32^. Taken together, these findings suggest that the interaction between CBD and opioids in modulating pain sensitivity may depend on the specific doses used, and may result in synergistic, additive, or subadditive effects. In the present study, we did not test the 30 mg/kg dose of CBD and used only a single dose of oxycodone for self-administration. Therefore, future studies are warranted to examine the effects of higher CBD doses and varying oxycodone doses in neuropathic pain models.

In this study, the effects of neuropathic pain, CBD, and oxycodone self-administration were examined only in male rats. Previous studies have shown that CCI of the sciatic nerve induces thermal hypersensitivity in both male and female rodents, with no significant sex differences observed^59,71^. However, in an acute pain model, intraperitoneal administration of CBD in naïve male and female rats revealed sex differences in antinociceptive effects as measured by the tail-flick assay. Specifically, only the highest dose (30 mg/kg) was effective in males, whereas females exhibited dose-dependent (0.3, 3, and 30 mg/kg) antinociceptive effects^31^. In females, these effects were also dependent on the estrous cycle, with significant antinociception observed during late diestrus but not during proestrus^31^. Additionally, in acute pain models, the antinociceptive effect of intraperitoneally administered oxycodone has been reported to be greater in females than in males in the tail-flick assay^68^. Moreover, previous studies have demonstrated sex differences in intravenous oxycodone self-administration in rats^62,72^. Therefore, further studies are warranted to investigate the effect of sex on CBD and oxycodone self-administration in neuropathic pain models.

This study has several limitations that should be considered. First, we did not assess the effects of CBD alone on nociception in either sham or CCI rats. As a result, it remains unclear whether CBD’s intrinsic antinociceptive properties contributed to the preserved analgesic effects during oxycodone self-administration. Second, we employed only a single unit dose of oxycodone (0.06 mg/kg/infusion), limiting our ability to determine whether neuropathic pain differentially modulates oxycodone reinforcement across a broader dose range. Pain-dependent differences in oxycodone intake may emerge at higher or lower doses. Third, the CBD dose range tested was restricted to low to moderate doses (1–10 mg/kg). Higher doses of CBD (e.g., 30 mg/kg), which have produced anti-allodynic effects in CCI models, were not examined^27^. Thus, the full CBD dose-response relationship and its interaction with oxycodone under neuropathic pain conditions remain to be defined. Fourth, nociception was evaluated solely using the Hargreaves test. Although this assay reliably measures thermal hyperalgesia, reliance on a single modality limits the ability to detect broader pain-related changes and potential CBD-oxycodone interactions. Prior work has shown that CBD can reverse mechanical allodynia, cold allodynia, and thermal hyperalgesia in neuropathic pain models, suggesting that CBD’s effects may manifest across multiple sensory dimensions^27^. Future studies using multimodal assessments (e.g., von Frey, cold allodynia, additional thermal tests) will be important to provide a more comprehensive characterization of CBD-oxycodone interactions. Fifth, this study was performed only in male rats. Previous studies have demonstrated marked sex differences in CBD-induced antinociception, oxycodone analgesia, and intravenous oxycodone self-administration^31,62,68,72^. Therefore, future work should explicitly incorporate sex as a biological variable. Finally, although not the primary focus of this study, the mechanisms by which CBD reduces oxycodone intake remain unresolved. CBD is known to act as a negative allosteric modulator at CB1 receptors, a 5-HT1A receptor agonist, and a modulator of GABAergic, dopaminergic, and opioidergic signaling, all pathways that could influence oxycodone reinforcement^28,64,73–75^. Additionally, converging in vitro and in vivo evidence indicates that CBD modulates nociception predominantly through TRPV1 activation followed by desensitization and through cAMP-dependent TRPV1 regulatory pathways^27,76,77^. These mechanistic observations, together with the behavioral limitations noted above, underscore the need for future studies incorporating full CBD and oxycodone dose-response curves, multimodal pain assays, and neuropharmacological analyses to determine how CBD influences both analgesia and reinforcement in chronic pain states.

​In conclusion, our findings demonstrate that CBD reduces oxycodone self-administration in adult rats, regardless of neuropathic pain status, and does not interfere with the antinociceptive effects of oxycodone in neuropathic pain. These results underscore the therapeutic potential of CBD for reducing oxycodone misuse while preserving its antinociceptive effects.

## Supplementary Information

Below is the link to the electronic supplementary material.

Supplementary Material 1

## Data Availability

Data are available from the corresponding author on request.
